# Tetrahydrocurcumin (THC) as a Melanogenesis Inhibitor in Melanoma Cell Lines

**DOI:** 10.1155/bri/6256669

**Published:** 2025-12-23

**Authors:** Yung-Shun Su, Kuan-Ting Lee, Yi-Chiang Hsu

**Affiliations:** ^1^ Department of Recreation and Sport Management, Tajen University, Pingtung 907, Taiwan, tajen.edu.tw; ^2^ Graduate Institute of Medicine, College of Medicine, Kaohsiung Medical University, Kaohsiung 807, Taiwan, kmu.edu.tw; ^3^ Department of Dermatology, Kaohsiung Medical University Hospital, Kaohsiung 807, Taiwan, kmuh.org.tw; ^4^ Department of Health and Beauty, Shu-Zen Junior College of Medicine and Management, Kaohsiung, 821, Taiwan, szmc.edu.tw; ^5^ School of Medicine, I‐Shou University, Kaohsiung 824, Taiwan

**Keywords:** melanin, reactive oxygen species (ROS), tetrahydrocurcumin (THC), tyrosinase

## Abstract

Melanin plays significant roles in biophysical, biochemical, light‐filtering, and cosmetic functions of the skin. The melanin content in pigmented cells, such as melanoma cells, can be accurately measured. This study focused on evaluating the effects of tetrahydrocurcumin (THC), a hydrogenated derivative of curcuminoids, on melanogenesis in melanoma cells. We specifically investigated how THC influences melanin production and tyrosinase activity, a key enzyme in the melanogenesis pathway, in A375 melanoma cells. Our results provide experimental evidence supporting THC’s potential as an inhibitor of melanin synthesis and tyrosinase activity. This suggests that THC could be an effective component in skin‐whitening products. By demonstrating THC’s ability to suppress melanin content and tyrosinase activity, our study highlights its potential for use as a dermatological agent in skincare formulations aimed at reducing pigmentation.

## 1. Introduction

Melanin is a natural pigment synthesized by melanocytes, primarily responsible for determining skin, hair, and eye color [1]. Beyond pigmentation, melanin plays a critical role in protecting the skin from ultraviolet (UV) radiation by absorbing and dissipating harmful rays, thereby reducing DNA damage and lowering the risk of photoaging and skin cancer [2, 3]. In this situation, the skin color darkens, and the overall antioxidant activity provided by melanin in that area increases, despite the melanin content per cell remaining constant [4]. Hence, under these conditions, the key factor is the melanin content or production per unit area, rather than the melanin content or production per individual cell [5].

The process of melanin biosynthesis, known as melanogenesis, is tightly regulated by several enzymes, of which tyrosinase is the most important rate‐limiting enzyme. Tyrosinase catalyzes the hydroxylation of L‐tyrosine to L‐DOPA and its subsequent oxidation to dopaquinone, leading to the formation of melanin [6–8]. Dysregulation of melanogenesis can result in hyperpigmentation disorders, which not only pose cosmetic concerns but also influence skin health. Because of the crucial role of tyrosinase in melanogenesis, inhibitors of this enzyme have been widely investigated as potential depigmenting and skin‐whitening agents [9, 10]. Current agents, such as arbutin [11], kojic acid [12], and vitamin C [13], are effective but often limited by stability issues, cytotoxicity, or poor bioavailability. Therefore, there is a growing demand for safer and more effective natural compounds that can both regulate melanin production and protect the skin from oxidative stress.

Tetrahydrocurcumin (THC), a major metabolite and hydrogenated derivative of the yellow curcuminoids found in the rhizomes of *Curcuma longa* [14, 15]. It has attracted attention due to its strong antioxidant and anti‐inflammatory properties, combined with its improved stability and lack of pigmentation compared with curcumin [15, 16]. Previous studies have shown that THC can scavenge reactive oxygen species (ROS) and protect against oxidative stress–related damage [17, 18]. Given the established connection between oxidative stress and melanogenesis, THC represents a promising candidate for developing skin‐whitening agents that combine antioxidant defense with melanogenesis inhibition. We aim to explore the in vitro effects of THC on inhibiting melanogenesis. THC’s lack of color and high antioxidant efficacy make it a valuable alternative in achromatic food and cosmetic products that traditionally use synthetic antioxidants.

Despite these advantages, the precise effects and mechanisms of THC on melanogenesis remain incompletely understood. Most previous reports have focused on curcumin or other derivatives [19, 20], whereas systematic investigations into THC’s role in modulating tyrosinase activity, melanin content, and ROS levels in melanoma cells are limited. To address this gap, the present study evaluated the effects of THC on melanogenesis using melanoma cell lines. We investigated THC’s influence on cell viability, melanin content, tyrosinase activity, and expression of melanogenesis‐related proteins, as well as its antioxidant activity. Our findings provide new insights into the potential of THC as a safe and effective natural agent for dermatological and cosmetic applications.

## 2. Materials and Methods

### 2.1. Materials

THC was obtained from Sigma‐Aldrich and dissolved in dimethyl sulfoxide (DMSO), with a final DMSO concentration < 0.1% in all experiments. DMSO and 3‐(4,5‐dimethylthiazol‐2‐yl)‐2,5‐diphenyltetrazolium bromide (MTT) were obtained from Sigma (St. Louis, MO). Cell culture medium (minimum essential medium), fetal bovine serum (FBS), antibiotics, sodium pyruvate, trypsin, and phosphate‐buffered saline (PBS) were purchased from Gibco, BRL (Grand Island, NY). Polyvinylidene fluoride membrane (PVDF) (Millipore) and molecular weight marker were purchased from Bio‐Rad (USA). All other reagents and compounds were of analytical grade.

### 2.2. Cells

The malignant melanoma (A375) (Number: 60263), amelanotic melanoma (C32) (Number: 60081), and human foreskin fibroblast (Hs68) (Number: 60038) cells were provided by the Bioresource Collection and Research Center (BCRC; Hsinchu, Taiwan). A375 cells (human malignant melanoma) were maintained on culture dishes in 90% Eagle’s Minimum Essential Medium with 2 mM L‐glutamine and Earle’s BSS adjusted to contain 1.5 g/L sodium bicarbonate, 0.1 mM nonessential amino acids, and 1.0 mM sodium pyruvate with 10% (v/v) FBS. C32 (human amelanotic melanoma) cells were cultured in 90% Minimum Essential Medium (Eagle) with 2 mM L‐glutamine and Earle’s BSS adjusted to contain 1.5 g/L sodium bicarbonate, 0.1 mM nonessential amino acids, and 1.0 mM sodium pyruvate. Hs68 (human foreskin fibroblast) was cultured in 90% Dulbecco’s modified Eagle’s medium with 4 mM L‐glutamine adjusted to contain 1.5 g/L sodium bicarbonate and 4.5 g/L glucose + 10% FBS. The cells were cultured in an atmosphere containing 5% CO_2_ in a 37°C incubator.

### 2.3. Cell Proliferation Assay

All cell lines were seeded into a 96‐well culture plate at 1 × 104 cells/well. The cells were treated with THC (0, 12.5, 25, 50, and 100 μM) for 1–3 days. MTT dye (1 mg/mL) was added to each well for at least 4 h of treatment. The reaction was stopped by the addition of 50–100 μL DMSO, and optical density was measured at 540 nm on a multiwell plate reader, following a previously described protocol [21]. Background absorbance of the medium in the absence of cells was subtracted. All samples were assayed at least in triplicate, and the mean for each experiment was calculated. Results were expressed as a percentage of the control, which was considered to be 100%, and the results were expressed as the mean ± SD.

### 2.4. Cell Cycle Analysis

The cells were treated with THC (0, 12.5, 25, 50, and 100 μM) for 24 h. Subsequently, the cells were harvested and washed with PBS by spinning at 300 g for 5 min at 4°C. Next, cells were fixed with 1 mL 70% ethanol at 4°C for at least 8 h. Afterward, ethanol was removed and the cells were washed with PBS. One milliliter of freshly prepared propidium iodide (PI) DNA staining solution (20 mg/mL) was used to resuspend the cells. The cells were kept in the dark at room temperature for 30 min. Stained cells were analyzed by flow cytometric analysis, following a previously described protocol [21]. DNA content was analyzed by flow cytometry analysis on a FACSCalibur system (BD, USA). At least 104 cells were analyzed for each determination. Data were analyzed by WinMDI 2.8 free software (BD, USA). The excitation/emission wavelengths were Ex 535 nm/Em 615 nm, respectively.

### 2.5. Evaluation of Apoptosis and Necrosis

The cells were first seeded in six‐well plates (Orange Scientific, EU). Following the treatment with THC for 4 h, the cells were harvested after the incubation period and washed in cold PBS. A 1× Annexin‐binding buffer (BD Pharmingen, BD, USA) and 100 μg/mL working solution of PI (Sigma, USA) were prepared. The washed cells were recentrifuged (the supernatant discarded) and resuspended in 1× Annexin‐binding buffer. 5 μL of FITC Annexin V (BD Pharmingen, BD, USA) and 1 μL of the 100 μg/mL PI working solution were added to each 100 μL of cell suspension, and the cells were incubated at room temperature for 15 min. After the incubation period, the stained cells were analyzed by flow cytometry and the fluorescence emission measurement yielded only low levels following a previously described protocol [21]. Apoptotic cells showed green fluorescence, and dead cells showed both red and green fluorescence. The excitation/emission wavelengths were Ex 488 nm/Em 550 nm, respectively.

### 2.6. Measurement of Melanin Contents

Melanin content was measured according to [22], with slight modifications to improve reproducibility in our laboratory. Briefly, B16F0 murine melanoma cells were seeded at a density of 1 × 10^4^ cells per well in a 24‐well plate. Cells were first stimulated with *α*‐melanocyte‐stimulating hormone (*α*‐MSH; Sigma) for 24 h to induce melanogenesis, followed by treatment with THC, vitamin C, or arbutin for 72 h. This sequential treatment differs from the simultaneous following a previously described protocol [23, 24] and was adopted in our study to stabilize baseline melanin production before compound treatment. After incubation, cells were washed with PBS, detached with trypsin‐EDTA, and collected by centrifugation. Cell pellets were photographed for qualitative assessment of pigmentation, solubilized in 1 M NaOH at 80°C for 60 min, and cooled to room temperature. The absorbance of the solubilized melanin was measured at 475 nm using a microplate reader (BioTek). Melanin content was normalized to total cell number and expressed as a percentage of control.

### 2.7. Measuring Tyrosinase Activity

The tyrosinase activity was determined by a previously described procedure with a slight modification [25]. Briefly, the incubation mixture contained tyrosine (1.38 mM, dissolved in a 67 mM phosphate buffer, pH 6.8) as a substrate, and 2 mL of the test compound solution was prepared at 37°C for 10 min. The reaction was initiated by the addition of 0.5 mL of tyrosinase (5 units/mL) at 37°C for 30 min, and then, the absorbance at 475 nm was measured with the ELISA reader (BioTek). The amount of dopaquinone was determined based on the absorbance. The following equation was used to calculate the inhibition of mushroom tyrosinase. Tyrosinase inhibition (%) was calculated as follows: % Inhibition = [(Ac − (At − Ab))/Ac] × 100, where Ac is the absorbance of the control (without inhibitor), At is the absorbance of the test sample, and Ab is the absorbance of the blank. Results are expressed as mean ± SEM of three independent experiments.

### 2.8. Cellular ROS Detection Assay

A375 melanoma cells were seeded at 1 × 10^5^ cells per well in six‐well plates and treated with THC (0, 12.5, 25, 50, and 100 μM) for 24 h. After treatment, cells were washed with PBS and incubated with 10 μM 2′,7′‐dichlorofluorescein diacetate (DCFDA; Sigma, USA) for 30 min at 37°C in the dark. DCFDA is a cell‐permeable fluorescent probe that, upon oxidation by intracellular ROS, is converted to the fluorescent compound DCF. After incubation, cells were washed, trypsinized, resuspended in PBS, and analyzed using a BD FACSCalibur flow cytometer. The excitation/emission wavelengths were Ex 488 nm/Em 525 nm, respectively.

### 2.9. 1,1‐Diphenyl‐2‐Picrylhydrazyl (DPPH) Assay

DPPH is a stable free radical with a red color (absorbed at 517 nm). According to Shimada et al.’s methods [26], which are based on the principle of scavenging DPPH radical, if free radicals have been scavenged, discoloration will occur and DPPH will change its color to yellow. Owing to this property of discoloration, this assay reflects the free radical scavenging activity of the analyzed herb (Molyneux P.). The stable free radical diphenylpicrylhydrazyl (DPPH) is used for estimating antioxidant activity [26]. THC was mixed with DPPH (0.1 mM) in ethanol solution, and after 20 min of incubation in the dark at room temperature, the absorbance was measured at 517 nm (against a reagent blank).

### 2.10. Western Blot Assay

A total of 50 μg of proteins were separated by 10% SDS‐PAGE and transferred to PVDF membranes (Millipore, USA). The membranes were blocked with blocking buffer (Odyssey, USA) overnight and incubated with anti‐*β*‐actin (Sigma‐Aldrich, St. Louis, MO, USA), anti‐glycogen synthase kinase‐3*β* (GSK3*β*), and antityrosinase (Santa Cruz Biotechnology, USA) for 1.5–2 h. The blots were washed and incubated with a second antibody (IRDye LI‐COR, USA) or conjugated with horseradish peroxidase (HRP) at a 1/20,000 dilution for 30 min. The antigen was then visualized using a near‐infrared imaging system (Odyssey LI‐COR, USA) or chemiluminescence detection kit (ECL; Amersham Corp., Arlington Heights, IL, USA). The data were analyzed using Odyssey 2.1 software.

### 2.11. Statistical Analysis

All data were reported as the mean (± SEM) of at least three separate experiments. A *t*‐test or one‐way ANOVA with post hoc test was conducted for statistical analysis, with significant differences determined as *p* < 0.05.

## 3. Results

### 3.1. The Effect of THC on Cell Proliferation

To determine the effect of THC on cell proliferation, A375, C32 melanoma cells, and HS68 (human foreskin fibroblast) were incubated with THC. All cells were treated with THC (0, 12.5, 25, 50, and 100 μM) for 1–3 days. Based on the results of the MTT assay, our data showed that THC has no cytotoxicity effects on cell proliferation in A375, C32 melanoma, and HS68 cells, respectively (Figure 1(a)). We have included representative microscopic images of A375 and C32 melanoma cells, as well as Hs68 fibroblast cells, treated with increasing concentrations of THC (0, 12.5, 25, 50, and 100 μM) for 24 h. As shown in Figure 1(b), THC treatment did not significantly affect the cell morphology across different concentrations, which is consistent with our MTT assay results showing no cytotoxic effects.

Figure 1The survival of melanoma cell lines (A375 and C32) and human skin fibroblasts (Hs68) was treated with THC. (a) An in vitro study was initiated by treating each of the melanoma and foreskin fibroblast cell lines with increasing doses of THC in A375, C32, and HS68 cells (0, 12.5, 25, 50, and 100 μM) for 24–72 h. An in vitro study was initiated by treating each cell line with increasing doses of THC for 24–72 h. The survival of these THC‐treated cells was then measured by the MTT method. (b) The representative microscopic images of A375, C32, and Hs68 cells, treated with increasing concentrations of THC (0, 12.5, 25, 50, and 100 μM) for 24 h. All images were captured at 20 × magnification (scale bar 20 m). Results were expressed as a percentage of the control, which was considered 100%. All data were reported as the means (± SEM) of at least three separate experiments.

**Figure:**
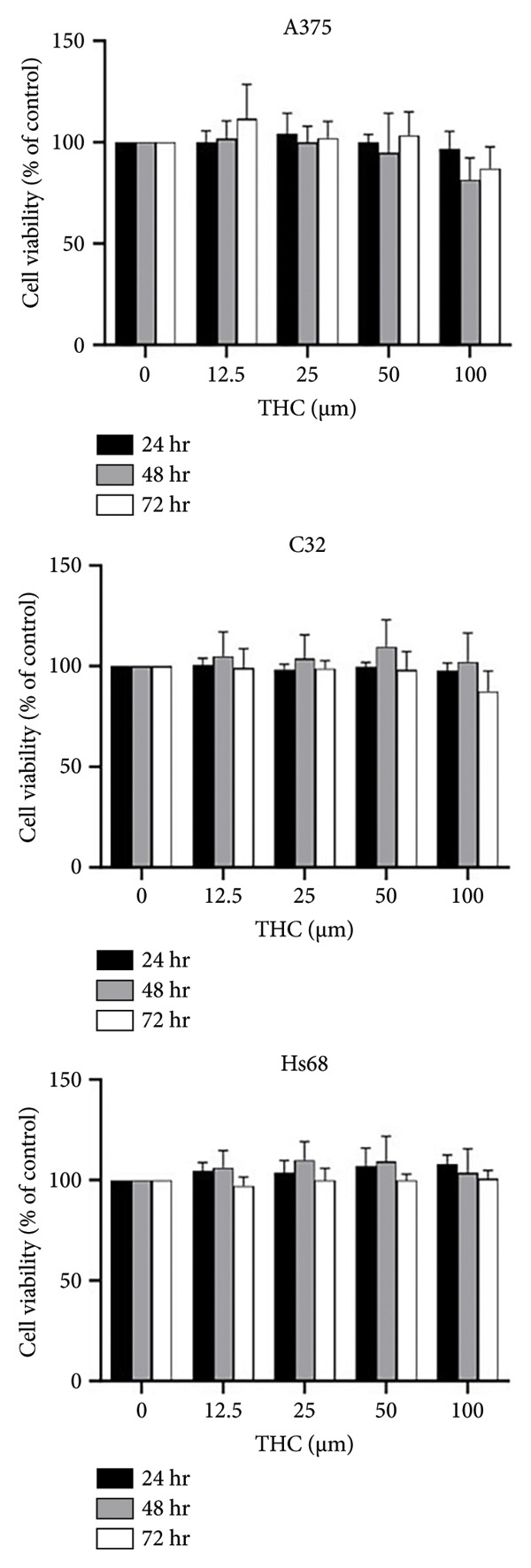
(a)

**Figure:**
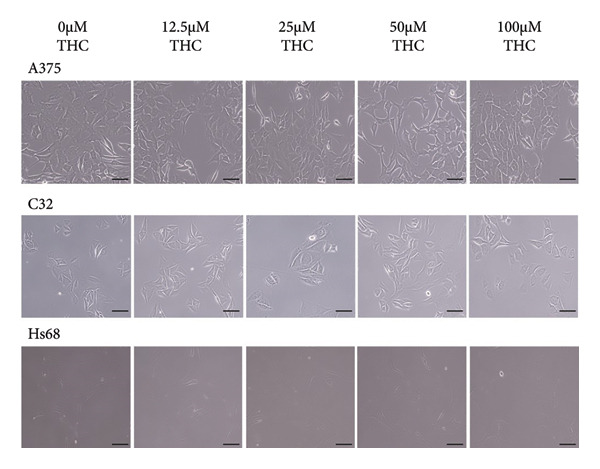
(b)

### 3.2. Nonapoptosis Induction in Melanoma by THC

To further elucidate whether THC exhibits effects on melanoma cells, we performed apoptosis/necrosis studies. After treating the cells with different doses of THC (0, 12.5, 25, 50, and 100 μM) for 4 h, the percentage of apoptotic/necrotic cells was assessed by Annexin V‐FITC and PI staining, followed by flow cytometric analysis (Figure 2(a)). The dot plot of Annexin V‐FITC fluorescence versus PI fluorescence also indicated a nonsignificant increase in the percentage of apoptotic cells that were treated by THC (Figure 2(b)). Furthermore, we also found that Hs68 (human foreskin fibroblast) does not induce apoptosis by THC treatment, which indicates that THC treatment does not cause cell death and apoptosis of normal cells.

Figure 2Total apoptosis in melanoma cells after 4 h of incubation with THC. (a) Melanoma cells were treated with THC (0, 12.5, 25, 50, or 100 μM) for 4 h and labeled with the dot plots of Annexin V‐FITC and PI. (b) The quantitative analysis of flow cytometry results. All data were reported as the means (± SEM) of at least three separate experiments.

**Figure:**
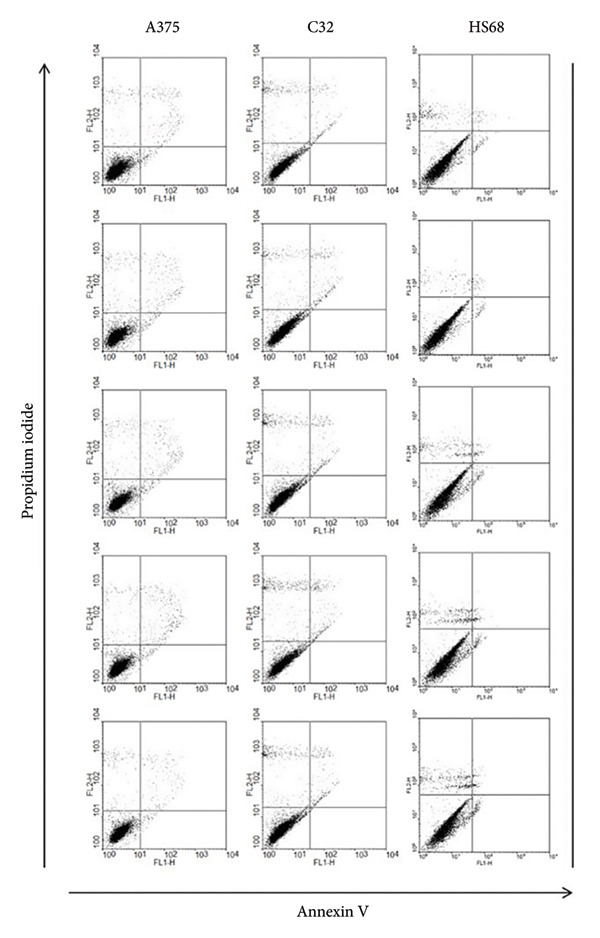
(a)

**Figure:**
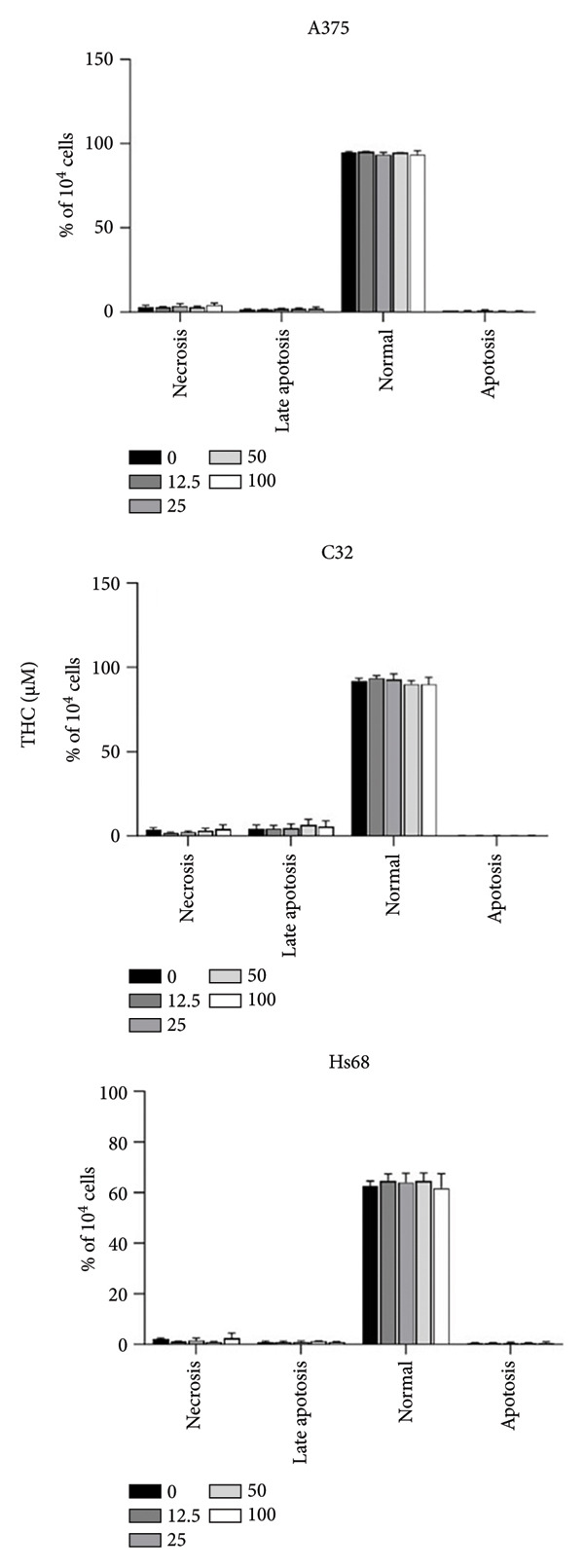
(b)

### 3.3. The Effect of THC on Cell Cycle

To explore the role of THC in affecting the cell cycle distribution in melanoma cells, the cell cycle distribution of THC‐treated melanoma and foreskin fibroblast cells was analyzed by flow cytometry. Before being processed and analyzed, the cells were exposed to THC for a total of 24 h. As shown in Figure 3(a), these concentrations had no effect on cell cycle, respectively. The cells exposed to THC showed no effect on cell cycle in A375 cells, C32 cells, and HS68 cells, as compared to that of the untreated cells in Figure 3(b). The observations may imply that THC has no effect on melanoma and foreskin fibroblast cells.

Figure 3Influence of THC on cell cycle progression/distribution in melanoma cells. (a) Cell cycle analysis of melanoma cells after being cultured with THC for 24 h. (b) To analyze DNA content, cells were dually stained using propidium iodide and quantified by flow cytometry. All data were reported as the means (± SEM) of at least three separate experiments.

**Figure:**
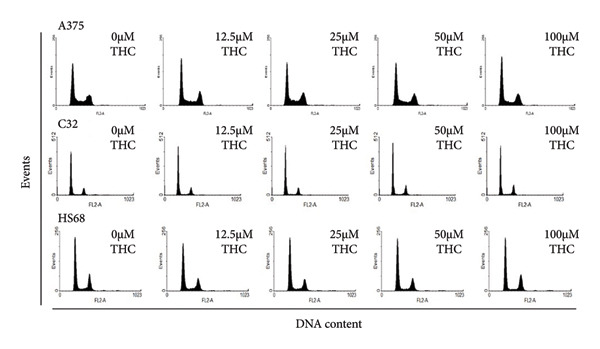
(a)

**Figure:**
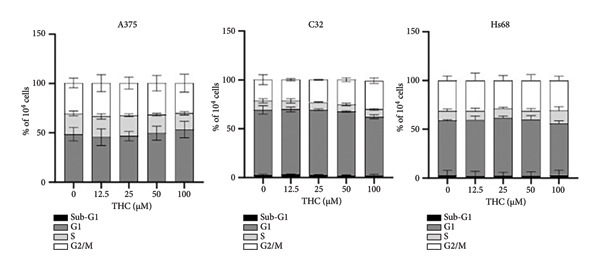
(b)

### 3.4. THC Inhibits Tyrosinase Activity Directly in Vitro

**Figure 4:**
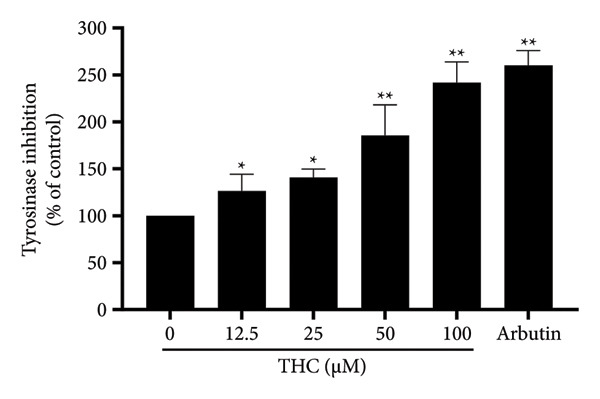
Tyrosinase inhibitory effects of THC in A375 cells. Arbutin 100 ppm was used/assigned as a positive control, and all data were reported as the means (± SEM) of at least three separate experiments, and arbutin 100 ppm is a positive control. A symbol (∗ or ∗∗) on the bars of each group indicates that the difference resulting from treatment with THC 0–100 μM is statistically significant at *p* < 0.05 or 0.01.

To further investigate the role of THC on tyrosinase expression in melanoma, we examined the inhibitory effect of THC on tyrosinase using mushroom tyrosinase. As shown in Figure 4(a), arbutin was used as a positive control group, THC showed inhibitory effects on tyrosinase, and inhibition was observed at concentrations of 12.5, 25, 50, and 100 μM, respectively. Our findings indicate that THC significantly inhibits the expression of tyrosinase activity in melanoma cells. THC may exert an inhibitory effect on tyrosinase activity, rather than suggesting an effect on the expression of tyrosinase activity.

### 3.5. DCFDA Cellular ROS Detection Assay

**Figure 5:**
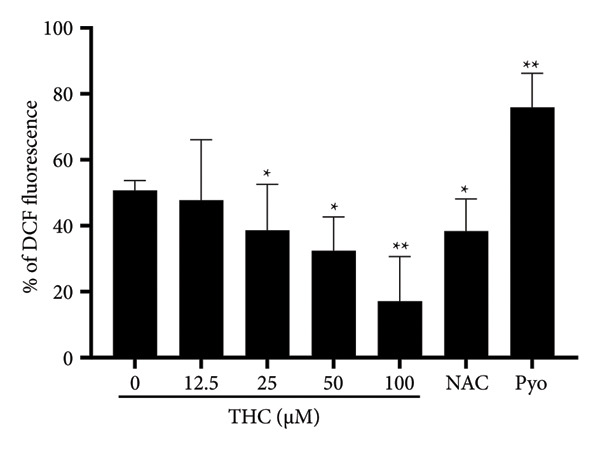
Effects of ROS inhibition by THC in A375 cells. Pyridine (Pyr) was used as a positive control, and all data were reported as the means (± SEM) of at least three separate experiments, and pyridine (Pyr) is a positive control. N‐acetyl‐l‐cysteine (NAC) was used as an ROS inhibitor and reported as the means (± SEM). A symbol (∗ or ∗∗) on the bars of each group indicates that the difference resulting from treatment with THC 0–100 μM is statistically significant at *p* < 0.05 or 0.01.

To confirm once again whether THC inhibited intracellular ROS, the cells were analyzed by flow cytometry using DCFH‐DA fluorescent dye. The oxidative stress effect was detected after THC treatment of melanoma cells to determine whether oxidative stress is produced in the cells. We evaluated the effects of oxidative stress and detected whether intracellular ROS were produced after the melanoma cells were treated with THC. The result showed that THC at concentrations ranging from 0 to 100 μM significantly reduced DCF fluorescence values in A375 melanoma cells in a dose‐dependent manner, indicating that THC effectively inhibits hydrogen peroxide‐induced ROS production. This effect was positively associated with increased intracellular NAC levels resulting from the inhibition of its efflux (Figure 5(a)). Results are positively related to NAC as a result of inhibition of the group, and findings show that as THC concentrations increase from 0 to 100 μM, A375 melanoma DCF fluorescence values markedly decreased and there is a dose‐dependent effect, showing that THC can inhibit the action of hydrogen peroxide in melanoma cells. In summary, our data suggest that THC significantly reduces the ROS expression in a dose‐dependent manner in melanoma cells.

### 3.6. DPPH Radical Scavenging Assay

Shimada et al.’s method [26] is based on the principle of measuring the scavenging ability of antioxidant substances toward 1,1‐diphenyl‐2‐picrylhydrazyl (DPPH), which is a stable free radical. The DPPH radical scavenging test is used to evaluate whether a component possesses antioxidant activity. 1,1‐diphenyl‐2‐picrylhydrazyl is a stable free radical, and at its maximum wavelength at 517 nm, DPPH can easily receive an electron or hydrogen from antioxidant molecules to become a stable diamagnetic molecule (reference). When the composition of the sample is directly added to the reaction with DPPH radical, the DPPH radical chain reaction is blocked, and the DPPH color changes from blue–purple to yellow. Using whitening substances, arbutin and vitamin C, as positive control groups, the data show that THC may be combined with the radical ion to inhibit the accumulation of free radicals in cells (Figure 6(a)).

Figure 6Inhibitory effects of THC on melanin production. (a) Free radical scavenging effects of THC in A375 cells. Vitamin C 100 ppm was used as a positive control, and all data were reported as the means (± SEM) of at least three separate experiments, and vitamin C 100 ppm is a positive control. (b) Arbutin and vitamin C were used as a positive control, and all data were reported as the means (± SEM) of at least three separate experiments, and arbutin and vitamin C were used as a positive control. A symbol (∗ or ∗∗) on the bars of each group indicates that the difference resulting from treatment with THC 0–100 μM is statistically significant at *p* < 0.05 or 0.01.

**Figure:**
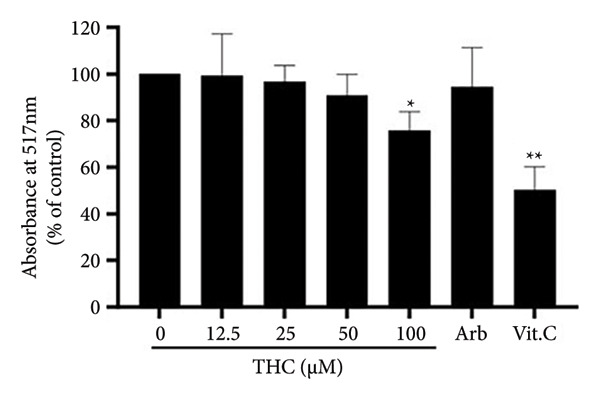
(a)

**Figure:**
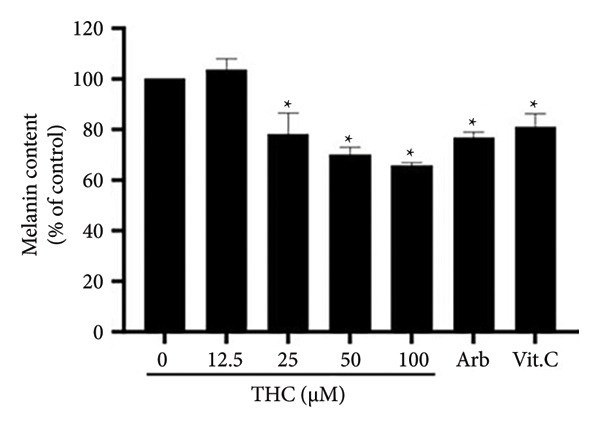
(b)

### 3.7. The Effect of THC on Melanin Production and Tyrosinase Expression

To assess the effect of THC on melanogenesis, *α*‐MSH was used and we quantified the melanin content in the THC‐treated B16F0 (nonmetastatic melanoma) cells. B16F0 murine melanoma cells were selected for melanin quantification due to their high pigment production and common use in melanogenesis studies, whereas A375 human melanoma cells were used for tyrosinase expression analysis to validate THC’s effects in human melanoma at the protein level. This complementary approach ensured that both functional and molecular aspects of melanogenesis were assessed. We use *α*‐MSH with the melanin contents of B16F0 melanoma cells decreased compared to the control (Figure 6(a)). The melanin contents of B16F0 melanoma cells treated with THC were expressed as a percentage of decrease/change relative to the control (Figure 6(b)). Among the *α*‐MSH‐stimulated cells, arbutin and THC were effective in whitening the cells. Although arbutin exhibited greater effectiveness than THC, the THC concentrations were lower than those of arbutin. In the presence of *α*‐MSH, melanoma cells were stimulated to enhance melanin synthesis. THC inhibited *α*‐MSH‐enhanced melanin synthesis in melanoma more significantly than other well‐known antimelanogenic agents such as arbutin and vitamin C.

### 3.8. The Effect of THC on Tyrosinase Protein Expression

Next, the immunoblotting showed that cellular proteins from A375 cells treated with THC revealed a decrease in tyrosinase after THC incubation, as shown in Figure 7(a). Quantification of tyrosinase, performed by measuring the relative band intensities, showed that tyrosinase levels were significantly lower in cells incubated with THC (Figure 7(b)) but not GSK3*β* activation in A375 cells treated with THC (Figures S1A and S1B). The results suggest that the level of tyrosinase inhibition in A375 cells has elevated (*y* = −8.6083*x* + 109.39; *R*2 = 0.9558) following the THC treatment for 24 h.

Figure 7THC represses tyrosinase gene expression in melanoma cells. (a) The cells were treated with THC (0, 12.5, 25, 50, and 100 μM) for 24 h, and tyrosinase gene expression was detected by Western blot analysis. (b) Quantification of band intensities. All data were reported as the mean (± SEM) of at least three separate experiments. Statistical analysis was performed using a *t*‐test, with significant differences determined at the level of ∗*p* < 0.05 versus the 0 μM control group.

**Figure:**
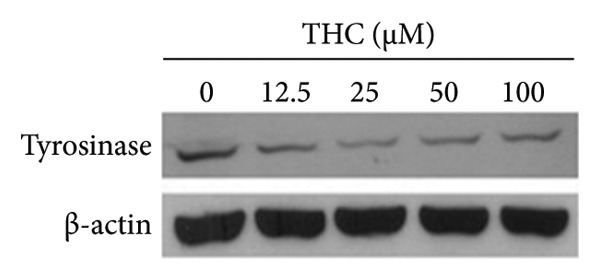
(a)

**Figure:**
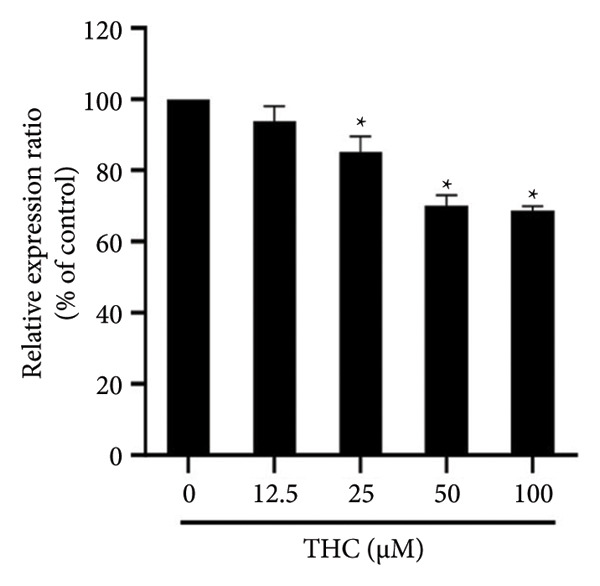
(b)

## 4. Discussion

The present study demonstrates that THC, a stable and colorless metabolite of curcumin, exerts dual antimelanogenic and antioxidant effects in melanoma cells without inducing cytotoxicity.

Melanogenesis in cultured pigment cells is determined not only by the production of melanin but also by the rate of cell growth. In stationary or senescent cells, the melanin produced accumulates within the cell and results in a rapid increase in melanin accumulation [27, 28]. In growing cells, the melanin is diluted in daughter cells during cellular division (dilution effect). If the melanin production rate equals the rate of dilution, the melanin content per cell will remain unchanged, as seen in the growing melanocytes [29, 30]. In rapidly proliferating melanocytes, when the rate of dilution exceeds that of melanin production, the melanin content per cell will decrease compared to the initial passages of melanocytes. However, the melanin content per cell remains constant when the cells are not actively dividing, such as in stationary cells [31, 32].

First, our findings confirm that THC is nontoxic to melanoma and normal fibroblast cells up to 100 μM, as shown by MTT viability assays, apoptosis analysis, and cell cycle profiling. This lack of cytotoxicity is critical for dermatological applications, as many skin‐whitening agents are limited by potential safety concerns.

Second, THC was found to directly inhibit tyrosinase activity, the rate‐limiting enzyme in melanogenesis. This result is consistent with previous reports showing curcuminoids’ ability to suppress tyrosinase [33]. Importantly, our study provides quantitative evidence that THC exerts significant inhibitory effects in a dose‐dependent manner, further validated by decreased tyrosinase protein expression in A375 cells.

Third, we demonstrated that THC reduces intracellular ROS levels in melanoma cells. ROS are well‐established mediators of melanogenesis, as oxidative stress can upregulate tyrosinase expression and melanin synthesis. The antioxidant effect of THC, as confirmed by DCFDA assays, highlights its dual functionality: not only as a tyrosinase inhibitor but also as a potent scavenger of oxidative stress, thereby enhancing its potential as a whitening and protective agent.

Fourth, THC significantly suppressed melanin production in *α*‐MSH‐stimulated B16F0 cells. Although arbutin and vitamin C showed stronger inhibition at equivalent doses, THC was effective at lower concentrations, indicating superior efficacy and suggesting potential synergy when combined with established whitening agents.

The nicotinic acid hydroxamate (NAH)–mediated increases in the phosphorylation of mitogen‐activated protein kinase (MEK)/ERK and AKT/GSK3β were also found in melanogenesis [34]. THC repressed the tyrosinase activity not through GSK3b activation in A375 cells. In the study of the effects of THC on melanogenesis of cultured pigment cells, cell growth rate may play a role in the results of melanin content per cell. A growth inhibitor that has no effect on the production of melanin per cell causes a decrease in cell number and a resultant reduction in melanin per cell due to the accumulation effect compared with the control (cultured without THC treatment). Based on the melanin content per cell, the effect of THC on melanin content could be evaluated as an inhibitor. Furthermore, it is important to assess whether THC treatment affects cell proliferation/apoptosis. THC’s lack of cytotoxicity is critical for its safe use in dermatological applications. The study showed no significant apoptosis or necrosis after THC treatment, confirming its safety in noncancerous skin cells [27]. This is consistent with other studies that found no cytotoxic effects of THC in various cell lines at concentrations suitable for therapeutic use [33].

Previous research on curcuminoids has demonstrated their effectiveness in inhibiting melanogenesis, and THC, being a more stable derivative, offers advantages such as better bioavailability and reduced cytotoxicity [35]. Arbutin and vitamin C are widely used for their skin‐whitening properties. In comparison, THC showed comparable results in reducing melanin content but required lower concentrations than arbutin [34]. This indicates that THC might be more efficient in lower doses, making it a valuable addition to cosmetic formulations.

Our findings demonstrate that THC reduces ROS levels. ROS are a significant factor in skin aging and hyperpigmentation. Excess ROS can lead to oxidative stress, which promotes the production of melanin and accelerates skin aging [35]. We demonstrated that THC significantly reduced intracellular ROS levels in melanoma cells, which aligns with previous findings on its antioxidant properties [36]. This reduction in oxidative stress further enhances its potential as a protective agent against environmental skin damage. Although this study provided valuable insights into THC’s effects on melanogenesis and ROS, further research is required to fully understand its molecular mechanisms, especially in in vivo models. Future studies should also investigate synergistic effects when combining THC with other known skin‐whitening and antioxidant agents. Exploring its potential for enhanced topical delivery, as shown in related compounds, could further increase its efficacy in skincare products [33, 37]. When compared to prior studies, our work expands current knowledge by integrating both functional assays (melanin content, tyrosinase activity, and ROS detection) and molecular validation (tyrosinase protein downregulation). Previous reports have emphasized delivery systems for THC [33, 37] or antioxidant properties [33, 37], but few have systematically evaluated its comprehensive effects on melanogenesis. Our findings therefore highlight the novelty of THC as a safe, stable, and dual‐action compound that combines antioxidant defense with melanin suppression.

Given that the biological and cosmetic impacts of melanin are influenced by the melanin content per unit area, the utilization of THC may result in a reduction in skin color darkness and an enhancement in the overall antioxidant capacity of melanin in that specific area (provided that the in vivo effect of THC parallels the in vitro findings). Therefore, the results collected in this series of studies provide experimental evidence supporting the contention that THC can be evaluated as a growth inhibitor and a mild melanin content repressing agent.

## 5. Conclusion

In conclusion, THC suppresses melanogenesis through direct inhibition of tyrosinase activity, downregulation of tyrosinase protein expression, and reduction in oxidative stress, all while maintaining minimal cytotoxicity. These properties underscore THC’s promise as a natural depigmenting and antioxidant agent for dermatological and cosmetic applications. Future in vivo studies and formulation research will be important to fully validate its clinical potential.

## Conflicts of Interest

The authors declare no conflicts of interest.

## Author Contributions

Conceived and designed the study: Yung‐Shun Su, Kuan‐Ting Lee, and Yi‐Chiang Hsu; statistical and biostatistics analysis of data: Yung‐Shun Su and Yi‐Chiang Hsu; writing, review, and/or revision of the manuscript: Yung‐Shun Su, Yi‐Chiang Hsu, and Kuan‐Ting Lee; supervision: Yi‐Chiang Hsu. All authors contributed to the design and implementation of the research, to the analysis of the results, and to the writing of the manuscript.

## Funding

This work was supported by the National Science and Technology Council (Taiwan; Grant no.: 113‐2314‐B‐037–110), I‐SHOU University (Taiwan; Grant nos.: ISU‐109‐IUC‐01, ISU‐111‐IUC‐08, and ISU‐114‐01–09A), and E‐Da Cancer Hospital (EDCHP112014, EDCHP112005, EDCHP113004, and EDCHP113005).

## Supporting Information

Supporting Information Figure S1: GSK3β protein expression in THC‐treated melanoma cells. (a) A375 cells were exposed to THC (0, 12.5, 25, 50, and 100) by Western blotting analysis. (b) Quantification of GSK3β activation showed that A375 cells treated with THC were not activated to GSK3β expression.

## Supporting information


**Supporting Information** Additional supporting information can be found online in the Supporting Information section.

## Data Availability

The data used to support the findings of this study are available in article supporting information.
