# Supplementary material for: Tetrahydrocurcumin (THC) as a Melanogenesis Inhibitor in Melanoma Cell Lines
Source: Biochem Res Int. 2025 Dec 23;2025:6256669. doi: 10.1155/bri/6256669 (PMC12723727; doi:10.1155/bri/6256669)
Supplement: Supplementary file 1 — Supporting Information Additional supporting information can be found online in the Supporting Information section. [file BRI-2025-6256669-s001.pdf]

Supplementary information

Figure S1. GSK3β gene expression in THC-treated melanoma cells. (A) The cells were treated with THC (0, 12.5, 25, 50 and 100 μM) for 24 h and GSK3 gene expression was detected by Western blot analysis. (B) Quantification of band intensities. All data were reported as the mean (±SEM) of at least three separate experiments. Statistical analysis was performed using a t-test.

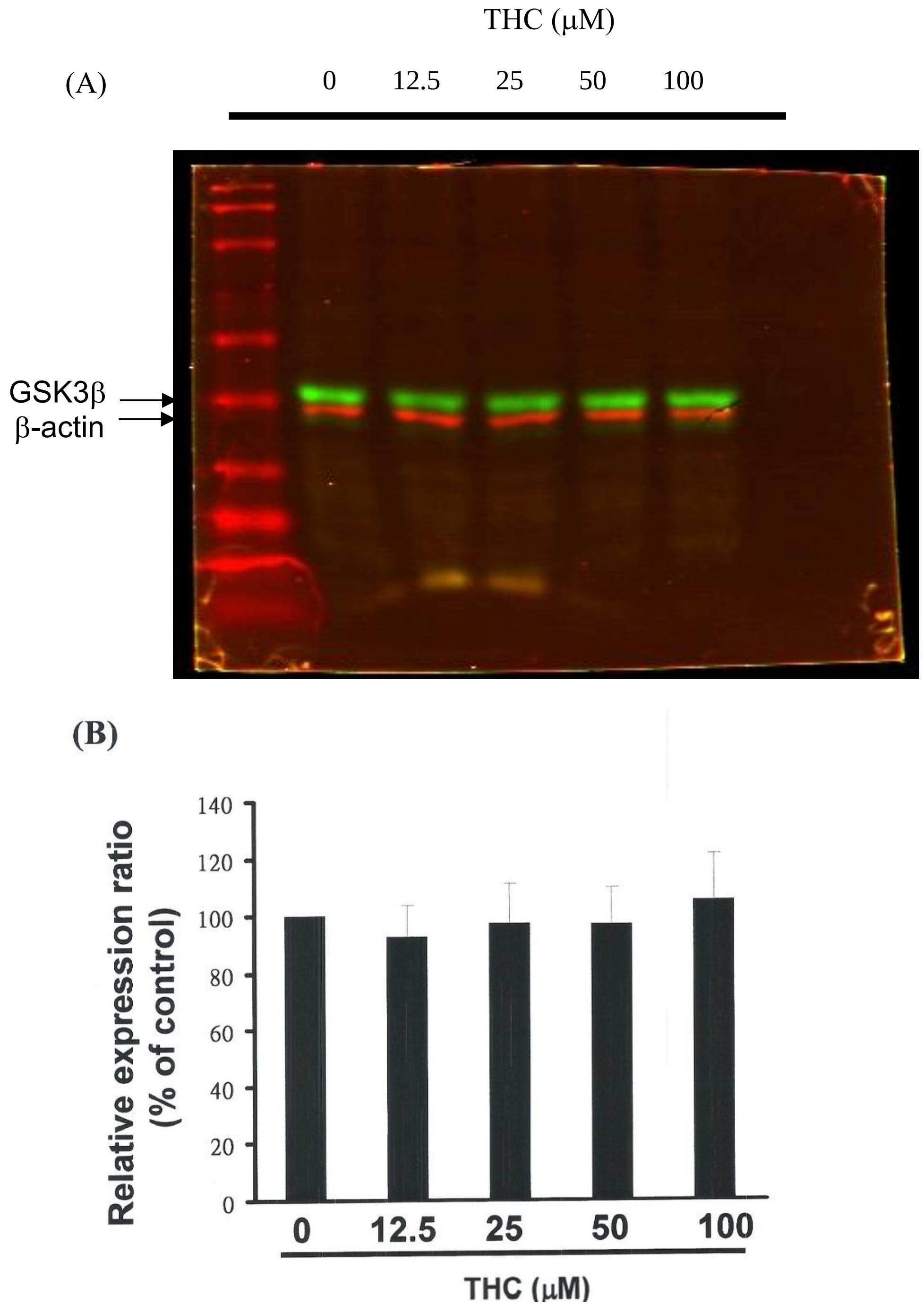

Western blot full gel

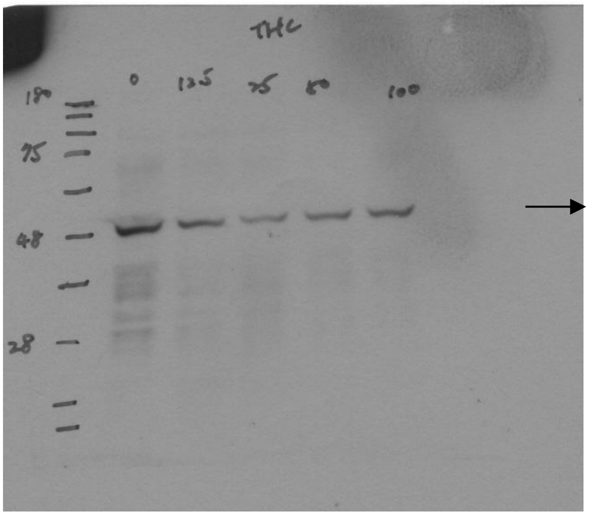

→ Tyrosinase

THC (μM)

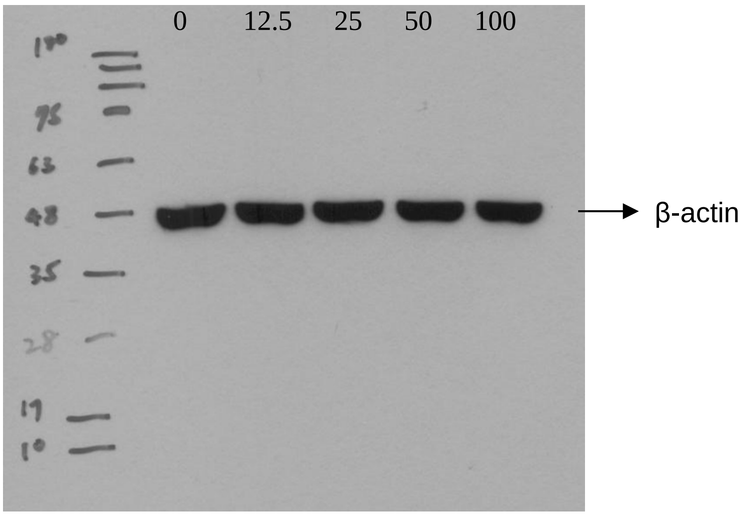

→ β-actin
